# Transcranial Magnetic Stimulation for Reducing the Relative Reinforcing Value of Food in Adult Patients With Obesity Pursuing Metabolic and Bariatric Surgery: Protocol for a Pilot, Within-Participants, Sham-Controlled Trial

**DOI:** 10.2196/50714

**Published:** 2023-11-06

**Authors:** Dale S Bond, Pavlos K Papasavas, Hollie A Raynor, Carlos M Grilo, Vaughn R Steele

**Affiliations:** 1 Department of Surgery Hartford Hospital/HealthCare Hartford, CT United States; 2 Department of Nutrition University of Tennessee Knoxville, TN United States; 3 Department of Psychiatry Yale University New Haven, CT United States

**Keywords:** obesity, repetitive transcranial magnetic stimulation, food reinforcement, hedonic hunger, electroencephalography, metabolic and bariatric surgery

## Abstract

**Background:**

Metabolic and bariatric surgery (MBS) is the most effective and durable obesity treatment. However, there is heterogeneity in weight outcomes, which is partially attributed to variability in appetite and eating regulation. Patients with a strong desire to eat in response to the reward of palatable foods are more likely to overeat and experience suboptimal outcomes. This subgroup, classified as at risk, may benefit from repetitive transcranial magnetic stimulation (rTMS), a noninvasive brain stimulation technique that shows promise for reducing cravings and consumption of addictive drugs and food; no study has evaluated how rTMS affects the reinforcing value of food and brain reward processing in the context of MBS.

**Objective:**

The goal of the *Transcranial Magnetic Stimulation to Reduce the Relative Reinforcing Value of Food (RESTRAIN)* study is to perform an initial rTMS test on the relative reinforcing value (RRV) of food (the reinforcing value of palatable food compared with money) among adult patients who are pursuing MBS and report high food reinforcement. Using a within-participants sham-controlled crossover design, we will compare the active and sham rTMS conditions on pre- to posttest changes in the RRV of food (primary objective) and the neural modulation of reward, measured via electroencephalography (EEG; secondary objective). We hypothesize that participants will show larger decreases in food reinforcement and increases in brain reward processing after active versus sham rTMS.

**Methods:**

Participants (n=10) will attend 2 study sessions separated by a washout period. They will be randomized to active rTMS on 1 day and sham rTMS on the other day using a counterbalanced schedule. For both sessions, participants will arrive fasted in the morning and consume a standardized breakfast before being assessed on the RRV of food and reward tasks via EEG before and after rTMS of the left dorsolateral prefrontal cortex.

**Results:**

Recruitment and data collection began in December 2022. As of October 2023, overall, 52 patients have been screened; 36 (69%) screened eligible, and 17 (47%) were enrolled. Of these 17 patients, 3 (18%) were excluded before rTMS, 5 (29%) withdrew, 4 (24%) are in the process of completing the protocol, and 5 (29%) completed the protocol.

**Conclusions:**

The RESTRAIN study is the first to test whether rTMS can target neural reward circuits to reduce behavioral (RRV) and neural (EEG) measures of food reward in patients who are pursuing MBS. If successful, the results would provide a rationale for a fully powered trial to examine whether rTMS-related changes in food reinforcement translate into healthier eating patterns and improved MBS outcomes. If the results do not support our hypotheses, we will continue this line of research to evaluate whether additional rTMS sessions and pulses as well as different stimulation locations produce clinically meaningful changes in food reinforcement.

**Trial Registration:**

ClinicalTrials.gov NCT05522803; https://clinicaltrials.gov/study/NCT05522803

**International Registered Report Identifier (IRRID):**

DERR1-10.2196/50714

## Introduction

### Background

Metabolic and bariatric surgery (MBS) is currently the most effective long-term treatment for obesity and its comorbidities [1-3]. However, there is substantial individual variability in the magnitude and durability of these outcomes, and patients who adopt and sustain healthier behaviors achieve better outcomes [3,4]. As a proximal behavioral driver of initial weight loss is reduced energy intake [5-7], there is a need to understand the mechanisms that can be targeted to promote healthier eating behaviors for improved MBS outcomes.

Food reinforcement is an important determinant of energy intake in the context of obesity and MBS [5,8]. Food is a potent primary reinforcer that motivates the initiation of eating [8,9]. By consuming a variety of foods, people learn which foods are pleasant and develop preferences influenced by the sensory qualities of these foods (eg, smell and taste) [8,9]. Eating preferred foods activates brain reward pathways and the release of dopamine that, over time via conditioning processes, promote greater wanting and intake of these foods in the absence of physiological hunger [8-15]. Thus, foods with higher reinforcing value are likely to be consumed more frequently and in greater quantities than foods with low reinforcing value.

Given that foods with higher reinforcing value also tend to be palatable and calorie dense, it is not surprising that higher food reinforcement is related to higher energy intake and obesity [8,16-23]. The reinforcing value of a food can be determined by how much work a person will do (or the number of responses they will make) to access that food [8,18]. The food reinforcer is provided on a progressive-ratio work schedule such that after a person earns a portion of the food, it becomes much more difficult to access the next portion. To better mirror eating in daily life, which involves making choices about whether, what, and how much to eat, the reinforcing value of food can be assessed by providing a choice to work for either a portion of a specified food or an alternative reinforcer such as money (ie, the relative reinforcing value [RRV] of food). The point at which a person makes the choice to switch from working for food to working for money serves as an index of the reinforcing value of food [8,18]. Research using RRV measures has found that people with obesity work harder for food and find food more reinforcing than nonfood alternatives compared with those who have a healthy weight [8,16,18-23]; higher food reinforcement predicts obesity severity and weight gain [8,16,18-24], and the relationship between food reinforcement and obesity is mediated by energy intake [25], suggesting that high food reinforcement leads to excess weight via energy intake.

Reduction in food reinforcement seems to be one of the ways in which MBS effects changes in energy intake [5,26,27]. MBS-induced anatomical and metabolic alterations are hypothesized to reset how rewarding food stimuli in the mesolimbic dopamine system are processed, leading to reduced hedonic hunger (eating for pleasure in the absence of physiological hunger) and related eating behaviors [14,26,28-30]. Support for this hypothesis is derived from studies showing postoperative reductions in questionnaire-based measures of food-seeking behavior and appetite for highly palatable foods as well as from progressive-ratio behavioral tasks of the reinforcing value of sweet and fat candy [27,31,32]. Furthermore, neuroimaging research shows the postsurgical normalization of obesity-induced alterations in brain reward regions (ie, caudate nucleus, putamen, nucleus accumbens, pallidum, and amygdala), improvements in overall functional connectivity, and increased activation of executive regions (ie, dorsolateral prefrontal cortex and ventral anterior cingulate cortex) during response inhibition to high-caloric food [14,28,33].

However, the MBS modulation of the mechanisms that influence food reinforcement is variable, with some patients being more resistant to these effects than others [34,35]. A characteristic of this *resistant* phenotype is greater hedonic hunger and susceptibility to overeating [31,35]. Moreover, changes in food reinforcement seem to be only temporary, with the re-emergence of unhealthy eating behaviors typically occurring approximately 2 years (but as early as 6 mo) after MBS [28,29,36,37]. Thus, strategies are needed that can directly target the neural mechanisms of food reinforcement, ideally before MBS, to prevent suboptimal outcomes.

Noninvasive brain stimulation interventions, such as repetitive transcranial magnetic stimulation (rTMS), are increasingly used to target dysregulated brain reward circuitry in individuals who have substance use disorders (SUDs) and in those who are prone to overeating [14,38-44]. rTMS exerts its neuromodulatory influence via electromagnetic coils that generate repetitive magnetic impulses to induce small electrical currents within a focal area in the superficial brain tissue below the scalp directly under the rTMS coil [43]. The main neural target of rTMS treatment for SUDs and overeating is the left dorsolateral prefrontal cortex (l-dlPFC), which drives mesolimbic dopaminergic regions to initiate motivated behavior [14,43,45]. In both SUDs and dysregulated eating, the l-dlPFC is hypoactive, contributing to heightened sensitivity to the reinforcing properties of substances and food and the failure of inhibitory control systems to resist temptation to consume them [14,43,45]. The application of excitatory rTMS to the l-dlPFC can upregulate neuronal excitability and alter synaptic plasticity to promote the lowering of the threshold of engagement of this region during exposure to drug and food reinforcers [38-45]. rTMS at this location could affect inhibitory control processes (ie, *top-down* mechanisms) or reward processes (ie, *bottom-up* mechanisms) because the l-dlPFC has structural connections to reward regions such as the dorsal and ventral striata [46]. These neuromodulatory changes, in the context of food reinforcement, could reduce motivated responding to food reinforcers and enhance eating regulation [43,44]. This empirical question has yet to be addressed.

Although a growing number of studies suggest that rTMS can be effective for reducing food cravings (ie, intense desire for a specific food), including among individuals with obesity [43,44], no study to our knowledge has directly examined the effects of rTMS on motivation to obtain a specific food that is reinforcing [8]. Unlike food craving measures, the reinforcing value of food directly measures motivation to eat and eating behavior by assessing how much work a person will do to obtain access to a palatable food. Furthermore, because the natural environment involves making choices between competing food and nonfood reinforcers, it is important to assess not only how much work a person will do to obtain a food but also how they choose to allocate work between reinforcing food and nonfood options [8].

### Objectives

Despite the potential benefit of rTMS for patients undergoing MBS, especially those who find food highly reinforcing and are at greater risk for overeating, no study has used rTMS in this clinical context. Thus, the *Transcranial Magnetic Stimulation to Reduce the Relative Reinforcing Value of Food (RESTRAIN)* study is the first to pilot-test the effects of excitatory rTMS applied to the l-dlPFC on food reinforcement using a validated RRV behavioral choice paradigm among patients who are pursuing MBS and have high levels of hedonic hunger. The aims are to compare the effects of active and sham rTMS on changes in the RRV of food via the behavioral choice task and the neuromodulation of reward via electroencephalography (EEG).

## Methods

### Ethical Considerations

The research and ethics described in this study have been reviewed and approved by the institutional review board (IRB) of Hartford Hospital (HHC-IRB 035431). The study protocol is registered at ClinicalTrials.gov (NCT05522803). All participants provide written informed consent after a thorough review of procedures and questions and are informed of their opportunity to opt out of the study at any time. All study data are deidentified. Participants are compensated at an hourly rate of US $20 for time spent in the laboratory and have the opportunity to earn an additional US $120 bonus for completing all study procedures.

### Design and Procedure Overview

**Figure 1:**
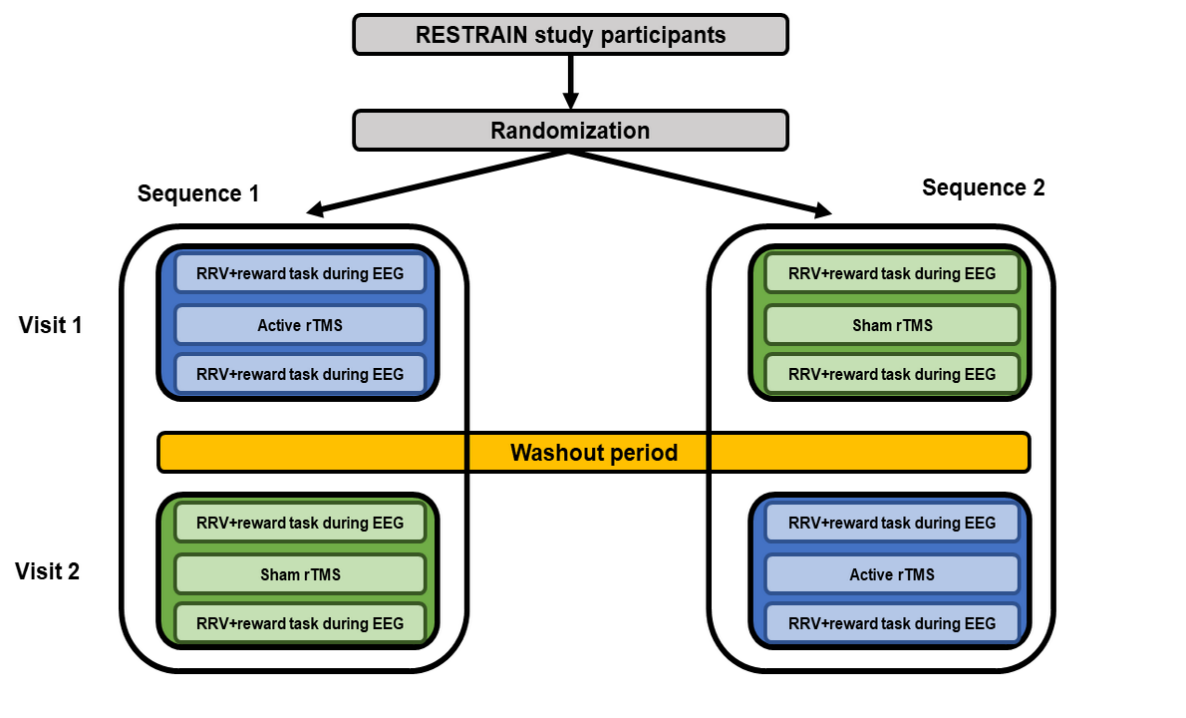
The Transcranial Magnetic Stimulation to Reduce the Relative Reinforcing Value of Food (RESTRAIN) study design. EEG: electroencephalography; RRV: relative reinforcing value; rTMS: repetitive transcranial magnetic stimulation.

A single-blind, within-participants, sham-controlled study is being conducted to perform an initial test of rTMS on the RRV of food, using a behavioral choice paradigm, and the neural modulation of reward, using an EEG (Figure 1).

Participants will attend 2 study visits that are separated by a washout period of at least 1 week (up to 4 weeks). Participants will receive active rTMS on 1 day and sham rTMS on the other day using a randomized and counterbalanced schedule. Participants will arrive at the laboratory fasted by 8 AM where they are asked to provide a urine sample for drug and pregnancy screening, complete an alcohol breath test, have their height and weight measured, and consume a standardized breakfast. During breakfast, participants will complete demographic, health history, and clinical behavioral and psychological questionnaires. After consuming breakfast, participants will complete a small sampling of 4 different palatable snack foods to determine which food will be used for the RRV measure for both study days. Participants will then complete the RRV measure and a reward task while an EEG is collected before rTMS (pre-rTMS EEG), receive rTMS, and complete the RRV measure and reward task again while an EEG is collected after rTMS (post-rTMS EEG). These procedures will allow for the comparison of pre- to posttest changes in the behavioral (RRV) and neural (EEG) modulations of reward between the active and sham rTMS conditions. Study procedures will be identical across study visits and conditions, except for certain baseline measures on the first day and the within-participant manipulation of active versus sham rTMS administration. A telephone follow-up to assess post-rTMS symptoms will be scheduled after the completion of each visit.

### Participants

Adults pursuing a primary MBS procedure at the Hartford Hospital surgical and medical weight loss center who are aged 18 to 60 years, are able to give valid informed consent in English, are without cognitive impairment, fulfill clinical criteria regarding hedonic hunger, meet safety criteria for an EEG and rTMS, and habitually consume breakfast within 3 hours of waking up will be eligible to participate in this study.

Patients will be ineligible if they have a history of neurological disorders that would increase seizure risk from rTMS (eg, stroke, previous neurosurgery, and head trauma resulting in a significant loss of consciousness); a first-degree family history of epilepsy, schizophrenia, bipolar disorder, or neurological disorders with a potentially hereditary basis that affect rTMS safety or EEG measures; cardiac pacemakers, neural stimulators, implantable defibrillator, implanted medication pumps or sensors, intracardiac lines, or acute and unstable cardiac disease, with intracranial implants (eg, aneurysm clips, shunts, and electrodes) or other metal objects in the body; current use of any investigational drug with anti- or proconvulsive action or medications with psychotropic effects (eg, benzodiazepines) for a disease that is not currently stabilized or with disease symptoms present; a lifetime history of schizophrenia, bipolar disorder, mania, or hypomania; a history of myocardial infarction, angina, congestive heart failure, cardiomyopathy, stroke, or transient ischemic attack; participation in any rTMS sessions ≤2 weeks before enrollment; current pregnancy; a history of self-reported hypoglycemia owing to diabetes in the last 3 months; and allergies to foods that are provided during the research visits.

### Screening, Recruitment, and Enrollment

Patients pursuing MBS will be recruited from the Hartford Hospital surgical weight loss center during the initial consultation visit for bariatric surgery. First, patients will receive an explanation of the study concept and a flyer from the surgeon during an office visit. Patients who are interested in being contacted by research staff for the study are asked to sign their name on the flyer and provided with a QR code to complete web-based screening questionnaires (refer to the following paragraphs). Patients will have the option of completing the screening questionnaires via REDCap (Research Electronic Data Capture; Vanderbilt University) at home on their own devices or over the telephone with a trained research assistant.

The screening measures that will be used to determine initial study eligibility are as follows:

1. *Power of Food Scale* (PFS) [47-49]: the PFS consists of 15 items, rated on a 5-point Likert scale, that assess preoccupation with an enhanced motivation to obtain and consume highly palatable foods across three separate but related domains—(1) food available, which assesses general thoughts about food; (2) food present, which assesses attraction to food that is directly available to a person; and (3) food tasted, which assesses desire for, and pleasure derived from, food when first tasted. PFS total and subscale scores are calculated by summing the item scores and dividing by the number of items. Higher PFS total scores relate to a higher drive to consume palatable foods. The PFS total score has good test-retest reliability, is internally consistent, is not affected by hunger states (consistent with the hedonic hunger construct), and is related to higher responsivity to food cues [47-50]. Given that there is no threshold to determine *high* PFS scores, we identify patients with high hedonic hunger levels as those who score ≥1 SD above the mean PFS total score (2.57, SD 0.45) obtained from a previous study involving a large clinical population of patients with obesity [48]. Thus, only patients who have a PFS total score of ≥3.00 are deemed initially eligible to participate.
2. *rTMS safety and appropriateness*: patients will be administered a questionnaire that the study team has previously used in clinical trials.
3. *Habit of eating breakfast*: patients will answer a question regarding whether they have a habit of eating breakfast in the morning within 3 hours of waking.
4. *Contact preference*: patients will indicate their preferred method of being contacted and provide permission to the research team to send the informed consent and Health Insurance Portability and Accountability Act (HIPAA) forms to their personal email account.

After patients complete the screening form, they will be contacted by the research assistant who will inform them of their eligibility, provide a short description of the study and answer any questions, confirm that they have no allergies to foods provided during the study visits, and schedule a remote verbal informed consent session using a hospital-approved Zoom (Zoom Video Communications, Inc) account.

The remote informed consent session will be conducted with the study research assistant, the patient, and a neutral witness who is not involved with the study. During the session, the research assistant will review the informed consent form with the patient in REDCap. If the patient is willing to participate, they will provide their verbal consent, which is documented on a progress note by the research assistant and the neutral third party. The research assistant and neutral third party will both sign hard copies of the informed consent and HIPAA forms. The patient will then be scheduled for their first study visit. When the patient arrives at the research center for this study visit, they will be asked to sign a hard copy of the informed consent and HIPAA documents.

### Study Visits

Each participant will attend 2 study visits (each approximately 7 hours in duration) that are separated by a washout period of at least 1 week (up to 4 weeks). Participants will arrive at the research center between 7 AM and 8 AM after an overnight fast with no food or drink after midnight. Participants will undergo height and weight measurement, provide a urine sample (for drug or pregnancy screening), and consume a standardized breakfast (choice of 3 flavors of a soft-baked breakfast bar with gluten-free options, 2 flavors of yogurt, and orange or apple juice) that is equivalent to 12% of daily caloric needs based on weight and age. Both the standardized breakfast, which is consumed before the first RRV measurement, and the standardized snack (also providing 12% of daily caloric needs), which is consumed before the second RRV measurement, will be provided to diminish the potential influence of physiological hunger or food deprivation and the palatability of foods consumed on food reinforcement measured via the RRV measure [8,51]. During breakfast, participants will complete questionnaires, including the PFS, the Three-Factor Eating Questionnaire [52] (this measure assesses 3 aspects of eating behavior: cognitive restraint [tendency to consciously restrict or control food intake], disinhibition [tendency to overeat in the presence of palatable foods or other disinhibiting stimuli], and hunger [susceptibility to feelings of hunger]), the Food Craving Inventory [53] (this measure assesses subjective food cravings and the consumption of particular foods), Daily Activity Behaviors Questionnaire [54] (this measure assesses time spent in sleep, sedentary behaviors, and physical activity in the past 7 days), rTMS safety screen (this measure assesses the appropriateness of administering rTMS) [55], Patient Health Questionnaire-9 [56] (this measure assesses the severity of depression symptoms over the past 2 weeks), Adult ADHD Self-Report Scale [57] (this measure assesses symptoms related to attention-deficit/hyperactivity disorder), Edinburgh Handedness Inventory [58] (this measure assesses dominant handedness), and the Wide Range Achievement Test-4 [59] (this measure assesses fundamental reading, spelling, and math skills).

After completing the questionnaires, participants will be asked to sample 4 different palatable snack foods (ie, Doritos nacho cheese–flavored tortilla chips [Frito Lay], Lay’s original potato chips [Frito Lay], Twix candy bars [Mars, Incorporated], and Chips Ahoy chocolate chip cookies [Mondelez International]) and rank their liking of each on a 100-mm visual analog scale anchored by *dislike very much* and *like very much* at either end. The food that participants rate as their most liked will be used for the RRV measure [60] at both study visits. After food sampling, participants will indicate their current levels of hunger and fullness using visual analog scales before being prepared for EEG procedures and completing the RRV measures and another reward task during the EEG procedure. Participants will consume a snack before undergoing rTMS to limit the potential effects of physiological hunger. After rTMS, they will complete the RRV and reward measures while an EEG is collected (RRV and reward measures as well as EEG and rTMS procedures are described in the following subsections). Participants will then be debriefed and monitored for any side effects, scheduled for their second study visit, compensated for their time, and discharged. After the washout period, participants return to the research center for their second visit. The washout period, identical testing procedures and environments, and counterbalancing procedures are intended to cancel out any carryover effects within the active and sham rTMS conditions. Procedures completed during the first study visit will be extended to the second study visit with the exception of randomization, height measurement, food sampling, and the completion of questionnaires used to determine study eligibility (ie, Patient Health Questionnaire-9, Wide Range Achievement Test-4, Adult ADHD Self-Report Scale, and Edinburgh Handedness Inventory). At the end of study visit 2, a brief rTMS blind assessment will be performed where the participant will be asked whether the rTMS session that day was active or sham. The participant will be asked to rate how confident they are in their choice on a scale ranging from 0 (*not at all confident*) to 10 (*very confident*).

### Primary and Secondary Outcome Measures

#### RRV of Food

The RRV of food will be measured by a validated behavioral choice questionnaire that asks participants to make a choice between receiving the food they rated as their most liked (ie, nacho cheese–flavored tortilla chips, original potato chips, candy bars, or chocolate chip cookies) and receiving money [60]. To determine how reinforcing food is in comparison with money, the behavioral choice questionnaire provides participants with 16 different choices, in which they make a choice between receiving the most liked food (100 kcal serving) and receiving the money (US $0.25). Each choice—food or money—is associated with a different number of button presses (using a tally clicker) required to gain access to the choice. Each of the 16 choices on the questionnaire requires the same number of button presses (ie, 20) for the money, whereas the number of button presses for the food increases with each choice. Choice 1 begins with 20 button presses for either the money or the food. The number of button presses required to receive the food increases in 20 response increments for choices 2 through 16. Thus, by choice 16, participants could have access to the food if they are willing to make 320 presses or receive the money for 20 presses. Participants will select whether they want money or food for choices 1 through 16. To produce valid responses, participants will be informed that they will be performing one of their choices by choosing 1 of 16 numbers from a hat, with the numbers representing the choice from the questionnaire (eg, if a participant randomly selects number 6, the participant will carry out the decision made for choice 6, which is either 120 button presses for the most liked food or 20 button presses for the money). After participants complete the number of button presses associated with the choice drawn, they will receive their choice (food or money). The reinforcing value of food is scored as the choice (1-16) when money is chosen instead of food. The higher the number associated with the choice of money, the higher the RRV of food. This measure has been shown to be valid and reliable for assessing the RRV of food [60].

#### Reward Task

Participants complete a version of the task described by Gehring and Willoughby [61] in which they choose between 2 monetary options (target stimuli) on each trial and then receive feedback indicating whether the choice resulted in winning or losing money on that trial. In this task, the target stimuli are 2 adjacent squares, each enclosing a number (5 or 25) representing a monetary value (in US cents). These stimuli remain on the screen until a choice is made between the left and right squares. Feedback stimuli follow the choice indicating the outcome of the participant’s decision, that is, the chosen box turns either red or green to signify either a win or a loss (with red or green as the winning color counterbalanced across participants), and the unchosen box turns the other color (either green or red) to indicate what the outcome of the trial would have been had that box been chosen. The feedback stimulus appears for 1000 milliseconds, followed by a blank screen for 1500 milliseconds preceding the onset of the next trial. All 4 possible combinations of 5 and 25, (ie, 5-5, 5-25, 25-5, and 25-25) are evenly crossed with the 4 possible win or loss outcomes (ie, win-win, win-loss, loss-win, and loss-loss), resulting in 16 trial types; thus, although the participant’s choice produces a designated outcome on each trial, signaled by the feedback, outcomes on future trials are not predictable from outcomes associated with prior choices (analogous to a roulette wheel or slot machine). Two sets of these 16 trial types, ordered randomly, are included in each block. Upon completion of a block, participants will receive feedback about their win or loss ratio within that block. The feedback received by the participant will elicit both a feedback-related negativity (FRN) and a reward positivity (RewP) when coupled with an EEG. The amplitude of the FRN largely indexes the relative loss presented in the feedback (ie, FRN amplitude is greater for trials where the participant loses 5 when the alternative was to gain 25 compared with losing 5 when the alternative was to gain 5). The amplitude of the RewP indexes the relative gain (ie, reward) presented in the feedback (ie, the RewP amplitude is greater in trials where the participant gains 25 when the alternative was to lose 25 compared with gaining 25 when the alternative was to lose 5). Extracting the underlying FRN theta (3-9 Hz) and RewP delta (<3 Hz) time-frequency power better measures these processes than amplitude alone.

#### EEG and rTMS Procedures

##### EEG Collection

For the EEG collection, participants are fitted with an elastic cap with embedded electrodes. These electrodes, once they are filled with gel, passively measure electrical brain signals during tasks at a very high resolution (5000 Hz). This high temporal resolution is the key advantage of an EEG as a measure, which produces robust and reliable brain measures across clinical populations. Measures will be collected using a BrainAmp MR Plus 64-channel electrode system (Brain Products GmbH) following standard manufacturer procedures. During data collection, participants will be seated in a comfortable chair 60 cm from the computer screen with access to a button-response box to perform the reward task.

##### rTMS Sessions

The rTMS sessions will be administered using a MagPro X100 including MagOption (MagVenture, Inc) stimulator equipped with a figure-eight coil. All sessions will be completed in the same laboratory that contains the EEG system used in this study [62]. During the rTMS sessions, participants will be seated comfortably in a chair and place their head on a chin rest for stabilization. Reducing head movement is essential for accurately placing and holding the rTMS coil during rTMS applications. Two separate coils that are similar in appearance and acoustic properties are available. One active unblinded coil will be used to determine the resting motor threshold (RMT); the other coil will be blinded (1 side active and 1 side sham) and used to deliver rTMS. The coils are calibrated quarterly against one another to ensure comparable output. Participants will be monitored throughout the study via staff interactions and a monitoring questionnaire assessing typical rTMS side effects (eg, headache).

After the scalp position closest to the motor representation (ie, the *motor hot spot*) is found, the RMT at this location will be determined. Using repeated single pulses, the coil will be moved to determine the optimal scalp position for producing visible contralateral movement in the first dorsal interosseous in the right hand. Pulses over this motor cortex location will be administered to identify the RMT using parameter estimation by sequential testing software [63]. The hot spot located during the initial rTMS session (study visit 1) will be saved in the neuronavigation system (Localite GmbH) and verified at the subsequent session (study visit 2), whereas the RMT will be measured from the hot spot at every session. In accordance with manufacturer instructions and accepted standards, hand motor cortex will be stimulated to obtain the RMT [64-66]. As the l-dlPFC is the target, the left hemisphere (right hand) will be used to determine the RMT.

The l-dlPFC will be targeted using the neuronavigation system (Localite GmbH) Montreal Neurological Institute coordinates (−50, 30, 36) thought to modulate the circuit implicated in reward processing. Over 2 sessions, participants will receive excitatory and sham rTMS sessions once in a single-blind fashion. Excitatory rTMS will consist of intermittent theta-burst stimulation parameters that include 3 pulses given at 50 Hz repeated every 200 milliseconds for a 2-second duration followed by 8 seconds of no stimulation. This sequence will be repeated for a total of 20 cycles, lasts 192 seconds, and delivers 600 pulses [67]. Magnetic field intensity will be set at medium intensity, gradually increasing to the goal of 100% of the participant’s measured daily RMT.

The coil will be set accordingly (active vs sham) for each study day with the order counterbalanced within participant. The blinded coil has 2 sides that can be placed on the participant’s head: 1 side active and 1 side sham (with shielding). These sides look identical to the research staff and the participant, but rTMS pulses are delivered only from the active side. The sham side of the coil is designed to mimic the auditory feedback and scalp pressure evoked by the active side of the coil. The MagVenture system also includes electrodes to be placed on the scalp, near the rTMS stimulation target. This scalp stimulation from the electrodes mimics rTMS administration (ie, causes similar discomfort) but does not modulate brain circuits as rTMS does, thus increasing the likelihood of maintaining the blind without affecting neural signals. The MagVenture sham system is the best commercially available system and can effectively mimic the discomfort of an active rTMS session. To avoid placebo effects, it will be emphasized to the participants that the sensation they feel is related to the stimulation of scalp nerves and muscles and that brain stimulation itself cannot be felt. Such procedures are effective in establishing these sham procedures [68]. Although the aforementioned efforts are taken to preserve the blind, it is possible that the participant will become unblinded to the condition during the study; therefore, a questionnaire assessing the blind and the pain felt will be administered to participants at the end of the study.

##### rTMS Safety Considerations

Although the anticipated risks and adverse events of rTMS are mostly low or minimal, life support equipment will be made available near the laboratory. All study team members who operate the MagVenture machine will have standard training for procedures, including training in rTMS device operation, supervised repeated practice in rTMS procedures, and testing for interrater reliability in RMT determination.

The rTMS monitoring questionnaire administration, staff observation, and interactions with participants will occur daily. If immediate medical intervention is required, the participant will be referred to an appropriate medical facility; an ambulance may be called as needed. Information on all adverse events will be recorded and reported to the IRB with each continuing review application. Unexpected or serious adverse events will be promptly reported to the IRB. Standard seizure monitoring procedures will be in place [69], and video recordings will be collected during rTMS sessions to help evaluate the session as needed for training or a review of adverse events. In the event a participant reports an rTMS-related symptom (most often, headache), the physician on the protocol will be consulted. If deemed necessary by the physician, a single dose of acetaminophen will be administered.

### Statistical Analysis Plan

The statistical analysis will be conducted using Stata 18 (StataCorp LLC). All continuous measures will first be assessed for their distribution characteristics through the use of Shapiro-Wilk tests and the construction of histograms to determine whether assumptions are met for parametric analysis; if not, nonparametric alternatives will be used. Baseline clinical and demographic characteristics of participants will be assessed using descriptive statistical measures of central tendency and dispersion: means and SDs for those variables meeting assumptions for parametric analysis and medians, range, and IQRs for those not meeting assumptions. The primary outcome of changes in RRV (scores after rTMS−scores before rTMS) for both the active and sham conditions will be computed. Tests comparing the pre-post differences for the active and sham rTMS sessions will be conducted using 2-tailed paired *t* tests (if distributions are normal) or Wilcoxon signed rank tests if the assumptions of distribution are not met).

The EEG data, once collected, will be imported into the MATLAB platform (The MathWorks, Inc) for processing. Data will be filtered offline at 0.1 to 30 Hz and epoched (1000 ms before feedback to 2000 ms after feedback) to capture the FRN and RewP time windows and allow for time-frequency analyses (ie, reducing edge effects). Established preprocessing methods (eg, eye-blink correction, additional filtering, and bad-channel identification) will also be applied. Both theta (3-9 Hz) and delta (<3 Hz) power will be extracted and a principal component analysis applied. This data-driven approach helps separate meaningful segments of the time-frequency surface related to the FRN and RewP components. EEG data will be used to determine differences between active and sham rTMS on the neural modulation of reward [62,70,71]. Pearson and Spearman correlational coefficients will be used to evaluate the associations of clinical assessment measures with RRV and task-specific neural measures (eg, correlation between food craving scores and neural activation related to reward processing). We will control for any differences in hunger and fullness ratings before each RRV task in analyses examining changes in RRV.

We do not anticipate any period effects (ie, when the outcome of interest changes with time irrespective of treatment effect) because the condition of the treatment is stable for both active and sham rTMS. Regarding carryover effects, we anticipate that the effect of a single session of rTMS will last no more than 24 hours (the washout period is >5 times the anticipated duration of effects).

Regarding participant and rTMS operator ratings of active versus sham rTMS, we will report descriptive data on the percentage of participants and operators who correctly identified the active condition. Such reporting on the success of blind manipulation is standard in the rTMS field.

### Sample Size and Power Considerations

As this is an initial proof-of-concept pilot study, the sample size was based on guidelines for pilot studies and by practical considerations. Guidelines for an appropriate sample size for a 1-group pilot study suggest 10 to 12 participants [72,73]. Assuming 20% study attrition, we will enroll 12 patients to achieve an analyzable sample of 10 (83%) patients. The effect sizes found will be calculated and appropriate power calculations performed to determine the sample needed to fully power a subsequent study.

## Results

Study recruitment began in December 2022. As of October 2023, a total of 52 patients have been recruited and screened, of whom 36 (69%) screened positive, and 17 (47%) were enrolled. Of these 17 patients, 3 (18%) withdrew before receiving rTMS, 5 (29%) withdrew after receiving rTMS, 4 (24%) are in the process of completing the protocol, and 5 (29%) completed the protocol. Analysis of data is planned for February 2024, with the manuscript expected to be submitted in April 2024.

## Discussion

### Principal Findings

Reduction in food reinforcement seems to be a principal way by which MBS lowers energy intake to promote weight loss and other health improvements [5,26,27]. However, the surgical modulation of the mechanisms that influence food reinforcement is variable, with some patients seeming to be more resistant to these effects than others [34,35]. This phenotype, characterized by high levels of hedonic hunger, can undermine MBS efficacy [31,35]. Patients pursuing MBS who demonstrate this high-risk eating phenotype may benefit from strategies that can directly target the neural mechanisms of food reinforcement.

This paper describes the protocol used in the RESTRAIN study. To our knowledge, this is the first study to perform an initial test of whether rTMS, a noninvasive procedure that delivers magnetic pulses to stimulate or inhibit nerve cells in the brain, can successfully target brain reward circuitry to diminish the reinforcing properties of food in patients pursuing MBS who are highly reinforced by food, more likely to overeat, and at risk for suboptimal surgical outcomes. Moreover, although previous studies have shown positive effects of rTMS on food cravings (ie, desire to consume a specific food) [44,45], this study is the first to directly examine the effects of rTMS on motivation to obtain and eat a well-liked food compared with a nonfood reinforcer using a validated behavioral choice task [8,60]. In addition, by measuring the acute rTMS-induced modulation of reward processing with EEG, this study has potential to provide novel insights into the neurobehavioral mechanisms of food reinforcement that can be targeted with rTMS and other interventions to improve eating regulation and weight outcomes after MBS as well as other obesity treatments. The data collected from this initial pilot trial will help determine the feasibility and acceptability of rTMS in patients pursuing MBS as well as an estimate of its effects on the RRV of food and EEG-measured reward processing. These data will be used to calculate the required sample size for a larger fully powered trial to test the effects of rTMS on eating regulation and relationships with weight change after MBS.

### Limitations

Although the use of noninvasive brain stimulation techniques such as rTMS in the context of MBS is highly novel and potentially beneficial, some patients may not be open to rTMS and may prefer other treatment options (eg, psychotherapy and medications). Even if rTMS reduces food reinforcement, the scalability of rTMS for this purpose is not clear because rTMS can be costly and needs to be administered by highly trained operators. Although this study involves only 2 study visits to determine whether rTMS has an acute effect on food reinforcement, it is likely that a fuller course of treatment, such as that which has typically been recommended for treatment-resistant depression (ie, several d/wk for 4-6 wk) is required to produce durable changes [74]. Finally, there is a potential that the results do not support our hypotheses. In this event, the data collected will still be valuable in demonstrating the feasibility of conducting rTMS in patients pursuing MBS and provide a rationale for additional studies to determine whether there is an optimal number of rTMS sessions and pulses as well as stimulation locations that can yield clinically meaningful changes in food reinforcement within this patient population.

### Conclusions

The RESTRAIN study is the first application of rTMS in MBS and the first study to use rTMS to target motivation to obtain food among people who have a strong drive to eat in response to the reward of palatable foods. Moreover, this study will measure the acute rTMS-induced modulation of brain reward processing with an EEG. If successful, the results would provide a rationale for a fully powered trial to test whether rTMS-related changes in food reinforcement translate into healthier eating patterns and improved weight and health outcomes after MBS. rTMS could potentially provide another treatment for patients who are experiencing suboptimal weight loss or significant weight regain owing to poor regulation of appetite and eating behavior.

If the results do not support our hypotheses, future studies will focus on whether it is possible to modify and refine rTMS to exert greater effects on food reinforcement and related outcomes.

## Abbreviations

EEG: electroencephalography
FRN: feedback-related negativity
HIPAA: Health Insurance Portability and Accountability Act
IRB: institutional review board
l-dlPFC: left dorsolateral prefrontal cortex
MBS: metabolic and bariatric surgery
PFS: Power of Food Scale
REDCap: Research Electronic Data Capture
RESTRAIN: Transcranial Magnetic Stimulation to Reduce the Relative Reinforcing Value of Food
RewP: reward positivity
RMT: resting motor threshold
RRV: relative reinforcing value
rTMS: repetitive transcranial magnetic stimulation
SUD: substance use disorder

## Data Availability

Data generated or analyzed during the study will be included in the published articles and their supplementary information files. Individual participant data that underlie the results reported in future published articles will be made available, after deidentification, to investigators whose proposed use of the data has been approved by an independent review committee identified for this purpose.
